# Effective Crack Detection in Railway Axles Using Vibration Signals and WPT Energy

**DOI:** 10.3390/s18051603

**Published:** 2018-05-17

**Authors:** María Jesús Gómez, Eduardo Corral, Cristina Castejón, Juan Carlos García-Prada

**Affiliations:** Mechanical Engineering Department, Universidad Carlos III de Madrid, 28911 Madrid, Spain; ecorral@ing.uc3m.es (E.C.); castejon@ing.uc3m.es (C.C.); jcgprada@ing.uc3m.es (J.C.G.-P.)

**Keywords:** bogies test rig, condition monitoring, crack detection, vibration analysis

## Abstract

Crack detection for railway axles is key to avoiding catastrophic accidents. Currently, non-destructive testing is used for that purpose. The present work applies vibration signal analysis to diagnose cracks in real railway axles installed on a real Y21 bogie working on a rig. Vibration signals were obtained from two wheelsets with cracks at the middle section of the axle with depths from 5.7 to 15 mm, at several conditions of load and speed. Vibration signals were processed by means of wavelet packet transform energy. Energies obtained were used to train an artificial neural network, with reliable diagnosis results. The success rate of 5.7 mm defects was 96.27%, and the reliability in detecting larger defects reached almost 100%, with a false alarm ratio lower than 5.5%.

## 1. Introduction

Effective crack detection in railway axles in service can avoid catastrophic failures that may cost human lives. Currently, railway axles in service are inspected using non-destructive testing (NDT) techniques, specifically ultrasound testing (UT), which is considered to be the most reliable solution. However, the main disadvantage of UT is that there is no available information on the status of the axle from one inspection to another. Thus, if propagation is fast, there is no possibility of avoiding fracture and its consequences for the whole unit. The definition of inspection intervals is a critical issue, since it must balance the risk of catastrophic failure versus the cost of the inspections and the withdrawal of trains during them. Determining inspection interval issues is not straightforward, and there is no standard procedure to follow. Generally, inspection intervals are established taking into account only if the vehicle is used for high speed or not. Other factors such as the application of the vehicle (motor or trailer) and the dynamic load (dependent on the railway line condition together with environmental conditions) could also be considered, as proposed in [1]. Moreover, UT does not present a good performance in changes of section, which are present in railway axles. The use of equipment is also critical, since manual inspections are still being used, and therefore human factors can affect the results.

The use of effective automatic online condition monitoring techniques would avoid the limitations of UT. The aim is to obtain features that can be used to clearly determine the status of the axle. The energy released when a crack appears or is growing can be detected using acoustic emission sensors. They have been widely used for this purpose, and they are a proper tool to detect cracks with active distress. Besides, when a crack appears, the behavior of vibration signals suffers a permanent change that can also be detected when the crack is inactive [2]. Vibration analysis has generally been accepted as the best tool for detecting all defects in rotating machinery. Vibration signals contain large information about the mechanical system. Depending on the type of defect, different types of test, vibration directions, and features have been proposed. More specifically, several state-of-the-art approaches to crack detection in rotors, showing the most commonly used methods, can be consulted in [2,3]. In spite of the efforts which have been made, this issue is not yet solved [4].

The size of vibration signals is large, so signal processing techniques must be used to easily handle the information and focus on the proper features to detect defects. A state-of-the-art of features selection for vibration-based condition monitoring can be found in [5]. Traditional methods are based on frequency domain techniques such as the fast Fourier transform (FFT). Recently, signal processing techniques that work in both time and frequency domains, such as the Hilbert–Huang transform (HHT) [6] or wavelet transform (WT) [7], have appeared and have shown better results. A review of the use of WT applied to vibration signals to find cracks in rotors can be found in [8].

In the work of Bustos et al. [9], it is proven that vibration signals can be used to find proper features that indicate the operation status of a high-speed train running in a gear operating state. In [10,11], vibration signals are used to detect defects on railway tracks. Specifically, Wei et al. [10] apply the wavelet packet transform (WPT) to process signals with good results.

In [12,13,14,15], it is shown that energy calculated by means of the WPT of vibration signals obtained in steady state is an effective feature to detect cracks in a scaled railway axle, using signals coming from a Machine Fault Simulator. In [12], cracks in the middle section of a shaft were effectively detected using the energy related to the 3× harmonic of rotating speed. The previous related work [13] shows that the same procedure may also be used to detect the location of the crack, showing that is possible to establish threshold values for WPT energies and distinguish different crack locations. In [14] it is shown that this technique can be automated using artificial intelligence techniques, specifically artificial neural networks (ANNs), with good results. ANNs have been widely used to automate fault diagnosis techniques, including applications for gearboxes [16], shafts [17], and bearings [18,19,20]. The work of Gómez et al. [15] shows that the energy calculated by means of WPT can also be applied to a full-scale railway axle. Clear changes were observed in some structural frequencies and in harmonics of the rotating speed. Up to now, all previous works had only measured the railway axle and the bearings in isolation, without the bogie.

The present work shows that vibration signals can also be used to detect inactive cracks in two railway axles with the bogie, tested on a rig. The induced cracks were performed in the middle section of the two axles. Vibration signals were processed using WPT energy. ANNs were used to automate the diagnosis of the status of the axle. The results are also used to discuss the most convenient vibration direction for this purpose.

## 2. Prototype Description

The bogie is a framework that carries wheelsets and a suspension system in railway vehicles. Wheelsets comprise the axle, two wheels and brakes. The bogie is connected to the axle by means of the axle boxes that contain a rolling bearing. The type of bogie tested was a Y21 Cse, which has two wheelsets and is commonly used as a trailer for freight transport.

General characteristics of Bogie Y21 Cse are shown in Table 1.

**Figure 1:**
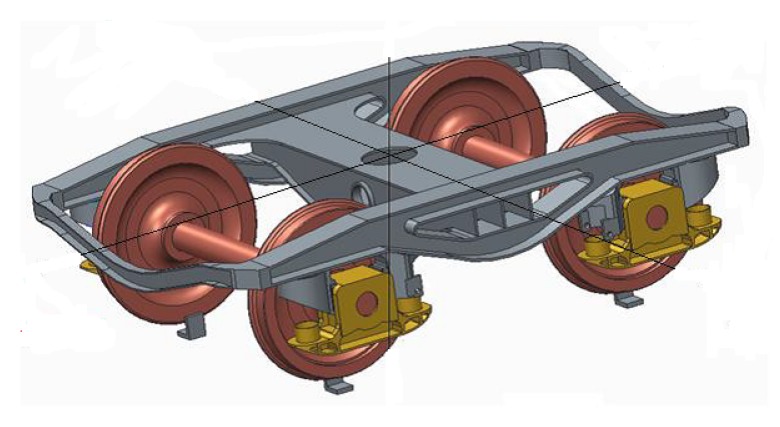
Bogie Y21 Cse.

A scheme of the bogie Y21 Cse is shown in Figure 1.

## 3. Experimental System Description

The bogie test rig, the measurement chain, the acquisition system, and the tests description are detailed in the following.

### 3.1. Bogie Test Rig

The bogie test rig is designed and manufactured by Dannobat Railway Systems. It is a machine specifically designed to test bogies with different defects in its elements. The aim is to obtain features that give information about the real status of the bogie.

**Figure 2:**
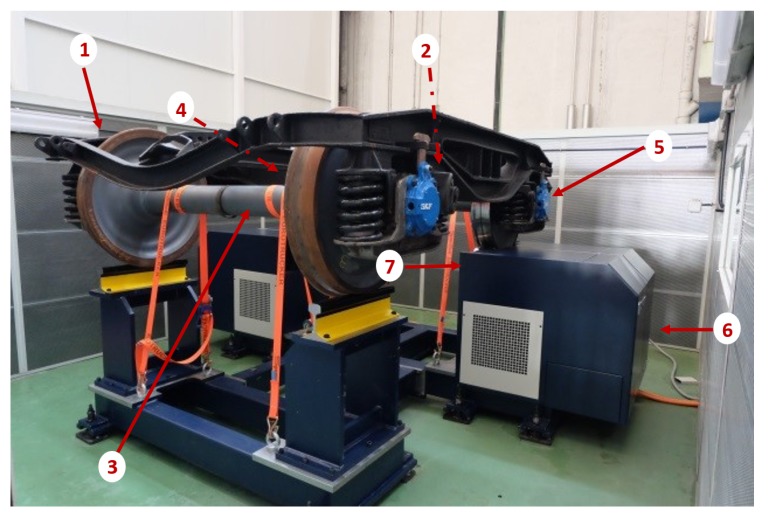
Bogie test rig with bogie Y21 Cse installed on it (**1**), tested wheelset (**2**), fixed wheelset (**3**), left-hand-side (LHS) axle box (**4**), right-hand-side (RHS) axle box (**5**), driving system (**6**), and driving roller (**7**).

The test rig is composed of a fixed bench and a drive system for rolling the axles. The drive system is controlled by the operator. One wheelset stands over the fixed bed, and the other is driven by rollers. The system drives the rollers, simulating the operating condition of the bogie. The test rig with the bogie is shown in Figure 2.

**Figure 3:**
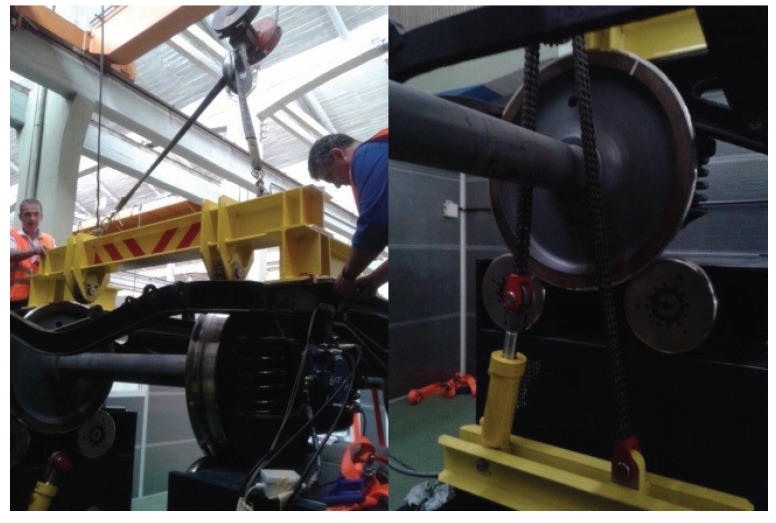
Bogie test rig load system.

Moreover, the test rig has a loading system that applies a vertical load on the bogie by means of hydraulic actuators. The maximum load applicable is 16 t. The load is transmitted through a chain that pushes a beam against the bogie. The load selected remains constant during the test, simulating real conditions. The loading system can be observed in Figure 3.

### 3.2. Measurement Chain and Acquisition System

**Figure 4:**
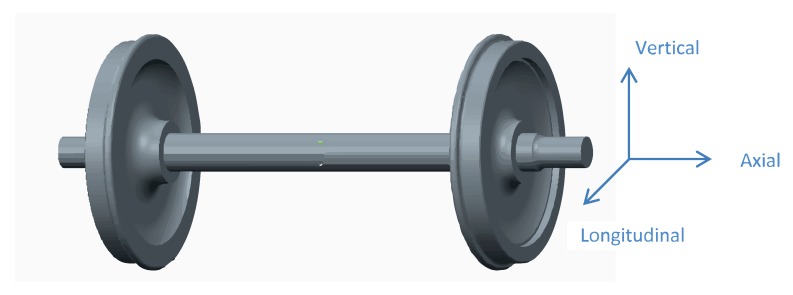
Wheelset and measurement directions.

The wheelset is supported by a pair of bearings included inside each axle box. Three uniaxial acceleration sensors were placed at each axle box of the wheelset (three on the right-hand-side (RHS) and three on the left-hand-side (LHS)) oriented in three directions: vertical, axial, and longitudinal, as defined in Figure 4. Thus, vibration signals from the bearing and from the wheelset were obtained.

**Figure 5:**
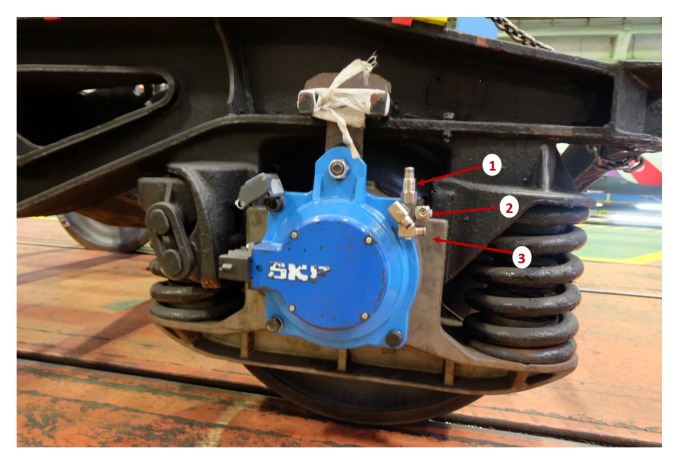
Sensors location with vertical (**1**), axial (**2**), and longitudinal (**3**) positions.

The sensors selected were Integrated Circuit Piezoelectric (ICP${}^{®}$) accelerometers of industrial use, model CMSS-RAIL-9100. They are commonly used in railway systems due to their technical characteristics, low price, and reduced size that allows easy installation. The sensitivity of the sensors is 100 mV/g, and the frequency range is 0.52 Hz–8 kHz. The location of the sensors is detailed in Figure 5.

The sensor was compatible with the conditioner system, SKF Multilog IMX-R, that was connected to a computer with the software SKF @ptitude Observer. This is a powerful and economic solution for railway vehicles and hard industrial environments. IMx-R complies with the Technical Specifications for Interoperability (TSI) for high-speed trains, and can include 24 inputs that can be measured at the same time. Data obtained were sent to a server where a SQL database was created. The acquisition system was designed to acquire 1.28-s signal samples when activated. The time selected is enough to measure several machine cycles, obtaining representative information. The parameters of the signal samples measured are shown in Table 2, chosen to avoid zero padding with a number of points power of two.

### 3.3. Tests Description

**Figure 6:**
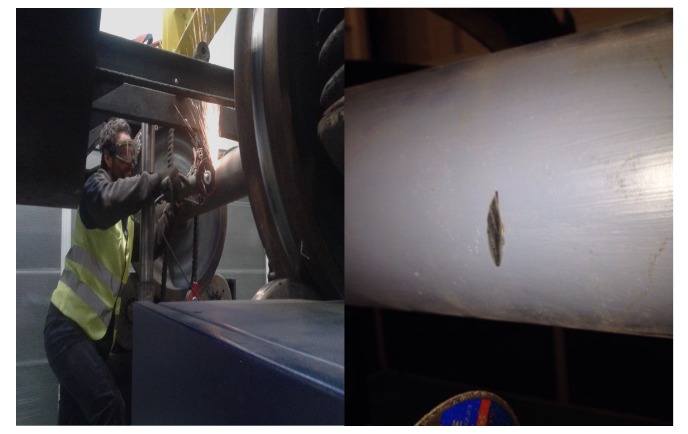
Inducing defects and result of Defect N1.

A total of two wheelsets, WS1 and WS2, were tested. Four different transversal crack levels were mechanically induced with a cutting tool, as shown in Figure 6.

The crack conditions tested are defined in terms of crack depth, and are shown in Table 3.

Given an installed wheelset with a certain induced crack level, a single test can start. Each single test was performed with a fixed value of load. The loads selected were 4, 10 and 16 tonnes. For every single test, different speed conditions were set in both rotation directions: clockwise (cw) and counterclockwise (ccw).

Before testing each wheelset, a running-in of grease was performed to make sure that the grease of the bearings was uniformly distributed. This first running-in of grease maintained the wheelset rotation for a whole week. Then, a daily running-in of grease was also performed before each single test to assure a constant temperature during the test and the normal operation of the system. No measurements were taken during the running-in period. Measurements were only taken at two different speed levels: 20 km/h (12.07 Hz) and 50 km/h (30.19 Hz). Each stage of the single test was established to take at least 60 signal samples, which is considered to be a statistically-representative group, and large enough to train an intelligent classification system. Therefore, a time of 28 min was enough to obtain the required signals. Thus, considering this set-up, a single test lasted approximately 4 h.

**Figure 7:**
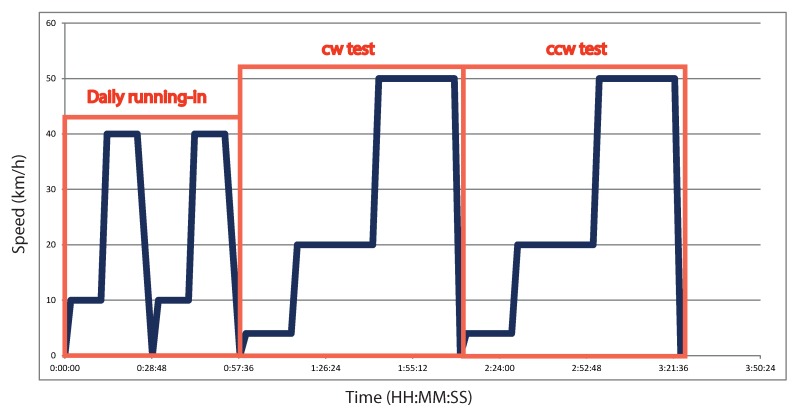
Detailed speed cycle for each single test. cw: clockwise; ccw: counterclockwise.

The speed profile versus time of a single test is detailed in Figure 7.

### 3.4. Data Processing

During the tests, all vibration signals obtained were stored in an SQL database. To process the signals using Matlab, it was necessary to create a connection between SQL and Matlab, allowing data conversion and extraction. A program developed in Matlab obtained the signals and classified them by groups of conditions.

**Figure 8:**
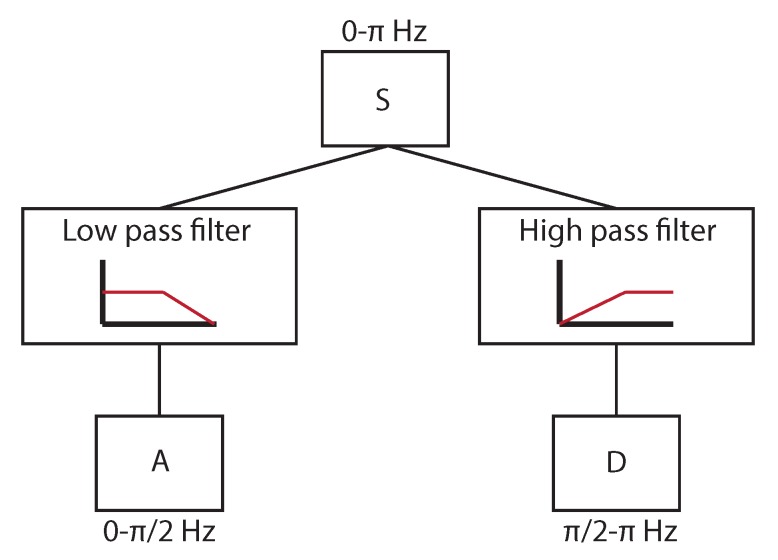
Example of decomposition in approximation (A) and detail (D) for a signal with a frequency range of π Hz.

All the signals obtained by all sensors in all directions and conditions were considered. All of them were processed by means of the WPT [21], which is one of the latest developments of the WT. WT calculates correlation coefficients resulting from the comparison of the analyzed signal with a wavelet function. The wavelet function is shifted and scaled, trying to maximize the correlation coefficients. The shift and scale parameters give information of both frequency and time domains, and thus transitory effects are considered. The WPT is a digital development of the WT, and allows the decomposition of a signal using filters that are related to the wavelet function. A low-pass filter and a high-pass filter decompose the signal, dividing the frequency range of the signal in two. The low-frequency information is called approximation (A), and the high-frequency information is called detail (D). The decomposition is shown in Figure 8.

**Figure 9:**
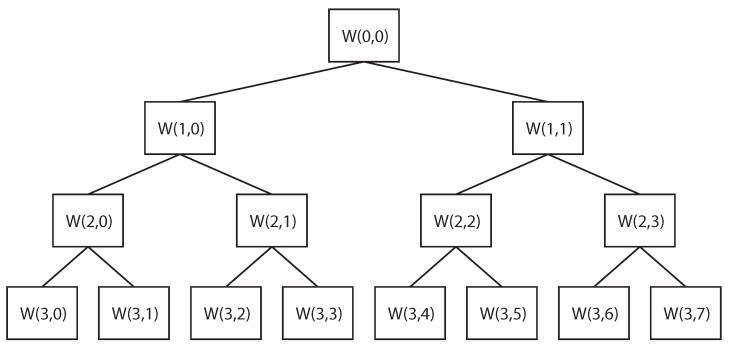
Example of wavelet packet transform (WPT) decomposition at level 3.

This decomposition was applied recursively to each group of coefficients obtained, called packets, until the desired decomposition level, as shown in Figure 9. At the end, all the packets obtained had the same frequency resolution.

W(k,j) is the vector of correlation coefficients of the signal in each packet, *k* represents the decomposition level, and *j* the position of the packet within the decomposition level. Each correlation vector has the structure shown in Equation (1):

(1) $$W\left(k,j\right)=\left\{w_{1}\left(k,j\right),...,w_{N}\left(k,j\right)\right\}=\left\{w_{i}\left(k,j\right)\right\}.$$

In this case, the wavelet function used was Daubechies 6. All signals were decomposed until decomposition level 6. Therefore, the number of packets obtained was 64 ($2^{6}$). Since the frequency resolution of the whole signal was 6.4 kHz (half the sampling frequency), the frequency range of each packet was 100 Hz. The packet number corresponds to the natural order of frequency ranges.

Later, the energy of each packet was calculated according to Equation (2):

(2) $$E_{k,j}=\sum_{i}{\left\{w_{i}\left(k,j\right)\right\}}^{2}.$$

## 4. Results

The main aim of this work was to check the reliability of the technique when diagnosing the different crack levels tested. It was convenient to find the best acceleration direction, load, and speed conditions for that purpose. The symmetry of the results obtained in both axle boxes (RHS and LHS) and in both rotation directions (cw and ccw) was also checked for both wheelsets. Thus, data were stored by groups of acceleration direction, load, speed, side, direction of rotation, and wheelset, and analyzed for each group.

First, correlation of the energy of packets with the crack depth was studied in order to find the packets that were more affected by the crack. This is called feature selection. The energies obtained were then used to train neural networks that would give diagnosis results.

### 4.1. Feature Selection

We studied the variation of packet energies with the crack depth when the rest of conditions were the same. The aim was to select the energy of packets that were influenced by the crack depth as features, and use them for crack detection. The energy distributions were supposed to have normal distributions (since we have at least 60 values for each). Thus, mean and standard deviation values were calculated by groups of conditions for each packet. Since the influence of the crack depth on the energy distribution may depend on the frequency (and thus on the packet analyzed) and on the test conditions, separate analyses were done. Several energy distributions of packets presented positive correlations with the crack size based on visual analysis (i.e., the energy increased with the crack depth). The energy of packets that increased with the crack depth at any case were selected by using visual analysis. The selected packets are shown in Table 4.

Packets 1 and 2 were related to the first harmonics of the rotation speed. The rest of features were related to structural frequencies of the bogie, where resonances occur.

The energy distributions for the selected packets were studied for all conditions. It was observed that the velocity and load affected the variation of energies. The best results were obtained at the conditions of 10 t–50 km/h, where a clear positive correlation between energy and crack depth was observed with symmetry for all packets selected as features. However, for the rest of the conditions, not all packets showed this correlation, or it was not observed with RHS–LHS and ccw–cw symmetry. This means that at other conditions, there may be some other phenomena that hide crack effects in some locations or conditions.

**Figure 10:**
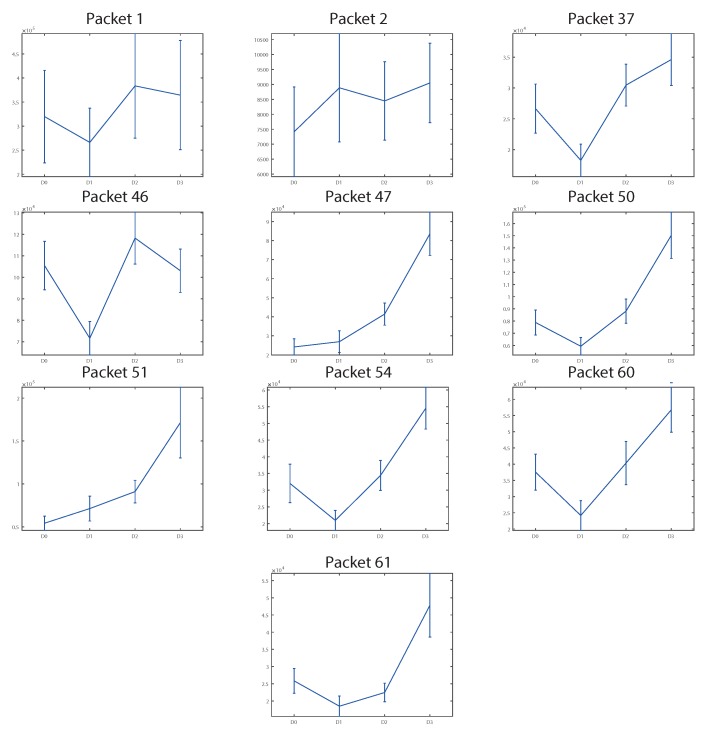
Evolution of energies (units: V${}^{2}/$s) versus crack levels D0, D1, D2, and D3 for features selected at WS2 LHS ccw 4 t–50 km/h at the vertical acceleration.

Figure 10 shows the energies in terms of mean and standard deviation versus the crack levels analyzed (the first one is D0, the healthy case) for the vertical direction at 4 t–50 km/h. The energies correspond to the LHS vertical sensor of WS2 rotating ccw. It can be observed that packets 1, 2, and 46 did not show any energy–crack depth dependence. The rest of the packets showed that the energy distributions were clearly higher for D3 than for D0, and they could be separated even visually. However, packets 31, 50, 54, 60, and 61 showed a decrease in energy for D1 with respect to D0 and erratic behavior for D2. For some packets, RHS–LHS and ccw–cw asymmetry was detected. Similar results were obtained for WS1.

**Figure 11:**
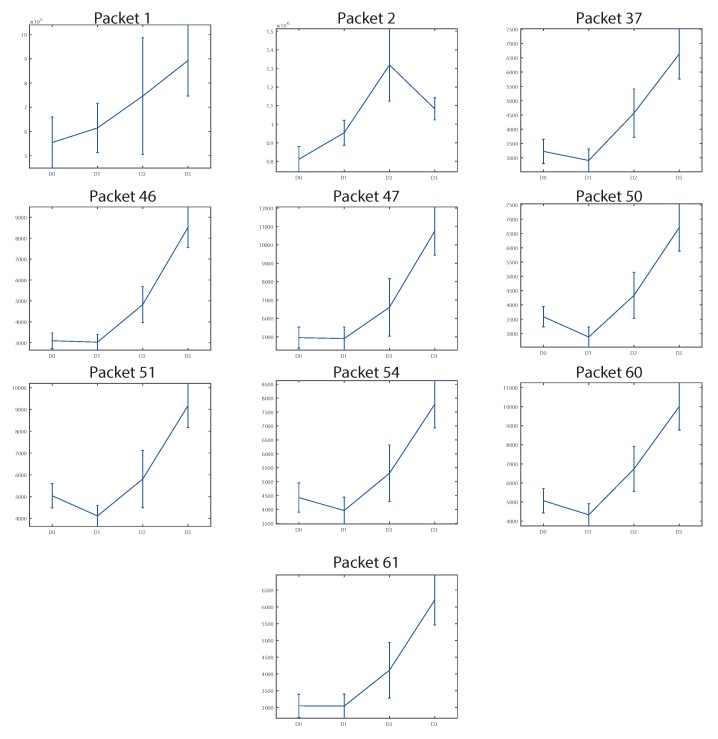
Evolution of energies (units: V${}^{2}/$s) versus crack levels D0, D1, D2, and D3 for features selected at WS1 RHS ccw 10 t–50 km/h at the vertical acceleration.

Figure 11 shows the energies versus the crack levels analyzed for the vertical direction at 10 t–50 km/h. The energies correspond to the RHS vertical sensor of WS1 rotating ccw. In this case, there was RHS–LHS and ccw–cw symmetry, and similar distributions were obtained for WS2.

It can be observed from Figure 11 that for all cases the energy distribution corresponding to the healthy case D0 could be clearly separated from the energy distribution of the crack level D3, even visually. Thus, these features can be used for crack detection, and at least crack level D3 can be diagnosed with high reliability at these conditions.

However, a combined analysis of the selected features may help to detect defects with reliability in all conditions—even D1 and D2 defects. Therefore, an intelligent classification system (i.e., neural networks) is used.

### 4.2. Neural Networks

The feature selection stage proves that the WPT energies processed are good features for crack detection. Thus, they can be used to train an intelligent classification system. For that purpose, radial basis function artificial neural networks (RBF-ANNs) with supervised training were used. The radial basis function selected was a Gaussian function.

Several trainings were done to find the best configuration for the RBF-ANN, with the aim of maximizing the reliability and minimizing the computational cost. After making several trainings of different RBF-ANNs considering separated acceleration directions, it was concluded that including the three directions did not involve a significant increase in computational cost, and diagnosis results were better, so a combination of the three directions was used.

The energies of the selected packets obtained at the three acceleration directions were used to feed the RBF-ANN. Besides, the values of speed and load were introduced. Thus, a total of 32 neurons at the input layer were used with normalized values. The supervised training was prepared to obtain two possible outputs: healthy or cracked. Seventy-five percent of the available data of each condition were used for training. The rest was used for the validation stage to obtain reliability results.

Classification system training had two stopping criteria established by the designer. The first one was related with the computational cost, and is set by means of the number of neurons in the hidden layer, since in the algorithm used creates a new neuron at each iteration. The other stopping criteria established was the mean squared error (MSE), also selected by the designer, and represents the error between the desired solution and the current solution.

The spread of the RBF-ANN must also be selected. This value represents the width of the Gaussian function and was selected by optimizing the results.

The design parameters for the neural networks trained are summarized in Table 5.

After selecting the parameters that optimize the results, the final RBF-ANN configuration is shown in Table 6.

The reliability results of this RBF-ANN are shown in terms of probability of detection (POD) curves. These curves show the probability for the response of the system to be “cracked” when the inputs are the 25% of the data of each condition kept for validation. The ideal response for D0 is 0%, which means false alarms, and 100% for the rest of the cases, which means success in diagnosis. The 95% of confidence limits were calculated. The lower limit was used for the case of defects D1, D2, and D3, and the upper limit was considered for the response of D0 or false alarms.

**Figure 12:**
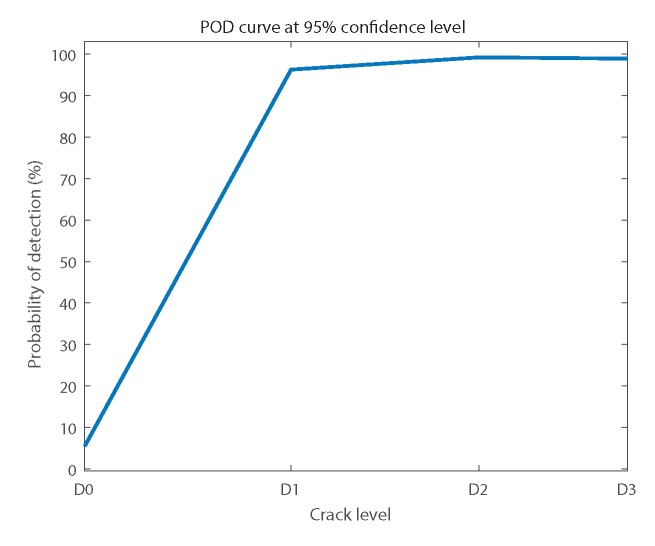
General probability of detection (POD) curve at 95% confidence.

Figure 12 shows the general POD that summarizes the reliability results, including all conditions tested.

It can be observed in Figure 12 that the diagnosis results were reliable. The false alarms value was at 95% of confidence lower than 5.48%. On the other hand, the success rate was at 95% of confidence above 96.27% for D1, 99.23% for D2, and 98.92% for D3.

**Figure 13:**
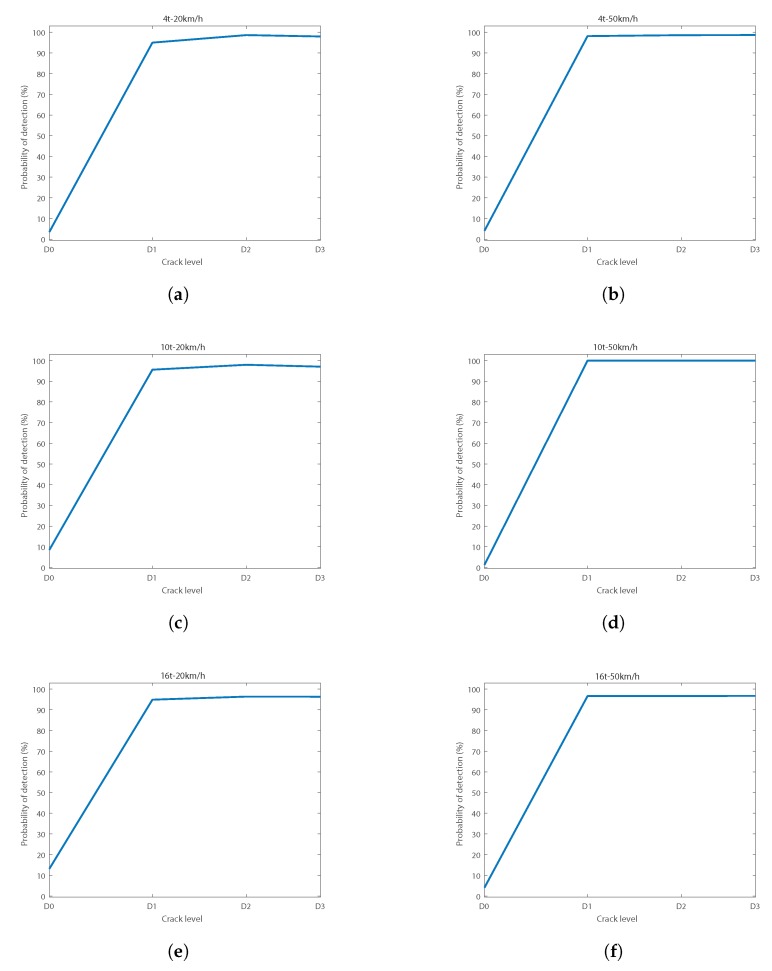
POD curves at 95% confidence for load and speed conditions at: (**a**) 4 t–20 km/h; (**b**) 4 t–50 km/h; (**c**) 10 t–20 km/h; (**d**) 10 t–50 km/h; (**e**) 16 t–20 km/h; (**f**) 16 t–50 km/h.

To find the best diagnosis conditions, the results were analyzed separately. Figure 13 shows the different curves obtained in the six different conditions of load and speed tested.

Regarding load and speed conditions, it can be observed from Figure 13 that the best diagnosis results were obtained at 10 t–50 km/h. At those conditions, the false alarms rate was under 1.28% at 95% confidence. Success rates when diagnosing a crack were above 99.97% for the three levels of crack tested (D1, D2, and D3). Attending to the speed, it can be observed that results were always better for the case of 50 km/h, where the false alarms rate was always lower than 5%. Additionally, results were better for the load of 10 t than for the cases of 4 t and 16 t.

## 5. Discussion

In previous related works working with scaled models, the effectiveness of the WPT energy of vertical vibration signals and RBF-ANN to diagnose transversal cracks was proven. In this work, the effectiveness of this combination working with a full-scale industrial bogie working on a rig was also checked.

In the feature selection stage, the correlation of energy with the crack depth was studied for different conditions. It was observed that all the features selected at the conditions of 10 t–50 km/h always showed a clear correlation with the crack depth. For the rest of the conditions, the correlation was not given in all cases, or it existed with RHS–LHS or ccw–cw asymmetry. With the idea of getting good diagnosis results with the combined analysis of all of the features selected, data were used to train several RBF-ANNs, selecting the best configuration. Feeding with all directions’ data, as well as load and speed values, the final RBF-ANN selected was able to diagnose the crack with high reliability for all conditions tested. However, the best results were obtained at 10 t–50 km/h.

Analyzing features, the vertical acceleration direction proved to have valuable information about cracks, since the correlation of the energy with the crack size was always observed. These features would allow crack detection with reliability. This behaviour was not observed for axial and longitudinal directions. Nevertheless, the trained RBF-ANNs were able to make better diagnosis working with data of all directions than using only the vertical direction. This allows us to conclude that all directions contain crack information. The constraints existing on the rig that are not present in real conditions can influence the results, but they exist in all directions. Signals coming from a bogie working in real conditions may show clearer crack information in the three directions.

In previous related works, it was found that increasing the speed improved the diagnosis results. This was also observed in the current work. Reliability was better at 50 km/h than at 20 km/h, possibly due to the better signal–noise ratio. However, further studies about the speed should be done.

The load influence was not studied in the previous related works highlighted, but the load does have an influence on the diagnosis results. For the three load conditions analyzed, the medium one showed the best results. The load seemed to amplify crack effects in vibration signals until a certain limit, above which increases in the load constrained and hid them.

## 6. Conclusions

In this work, a bogie test rig was used to extract vibration measurements with the aim of finding induced cracks in one of the axles of the bogie. Two complete wheelsets were measured, using six sensors on each. Three sensors were installed at each axle-box of the wheelsets tested, corresponding to vertical, longitudinal and radial directions. Measurements were taken at several conditions of transversal cracks, load, speed and rotation direction.

Vibration signals of the two wheelsets were processed using the WPT energy using Daubechies 6 as the wavelet function and decomposition level 6 (64 packets). WPT energies of all packets for all crack depths were studied. Under certain conditions, a clear positive correlation was observed for several packets related with harmonics of the rotation speed and with structural frequencies.

Data obtained from WS1 and WS2 were used to train several RBF-ANNs and select the optimal one. For this case, the results showed good reliability, especially for the case of 10 t–50 km/h, where the false alarms rate did not exceed 1.3%, and defects larger than 5.7 mm could be diagnosed.

## Figures and Tables

**Table 1:** Y21 Cse characteristics.

**Maximum load (t)**	60
**Maximum weight (t)**	20
**Brake**	Pneumatic
**Maximum speed (km/h)**	120
**Width (m)**	2.1
**Length (m)**	4

**Table 2:** Parameters of the signals measured.

Sampling Frequency Fs (kHz)	Acquisition Time for Each Signal (s)	Number of Points N
12.8	1.28	16,384 ($2^{14}$)

**Table 3:** Crack depths.

**D0**	0 mm (healthy)
**D1**	5.7 mm
**D2**	10.9 mm
**D3**	15 mm

**Table 4:** Packets selected as features and related frequency bands.

Packet Number	Related Frequency Band (Hz)
1	0–100
2	100–200
37	3600–3700
46	4500–4600
47	4600–4700
50	4900–5000
51	5000–5100
54	5300–5400
60	5900–6000
61	6000–6100

**Table 5:** Design parameters for the radial basis function artificial neural network (RBF-ANN). MSE: mean squared error.

**Number of neurons at input**	32	
**Normalization of input values**	Between [−1;1]	
**Number of neurons at output**	1	
**Normalization of output values**	[−1,1]	
**Input data distribution**	Training	75%
	Test	25%
**Stopping criteria**	*MSE*	0.1–0.2
	Number of neurons at hidden layer	700
**Spread**	0.2–2	

**Table 6:** Parameters of the signals measured.

Spread Value	Neurons at Hidden Layer	MSE
1	201	0.1
